# Quantum-dot-based suspension microarray for multiplex detection of lung cancer markers: preclinical validation and comparison with the Luminex xMAP^®^ system

**DOI:** 10.1038/srep44668

**Published:** 2017-03-16

**Authors:** Regina Bilan, Amagoia Ametzazurra, Kristina Brazhnik, Sergio Escorza, David Fernández, María Uríbarri, Igor Nabiev, Alyona Sukhanova

**Affiliations:** 1 Laboratory of Nano-Bioengineering, National Research Nuclear University MEPhI (Moscow Engineering Physics Institute), 115409 Moscow, Russian Federation; 2 Department of Research and Development, Progenika Biopharma S.A., Derio, 48160 Spain; 3 Laboratoire de Recherche en Nanosciences, LRN - EA4682, Université de Reims Champagne-Ardenne, 51096 Reims, France

## Abstract

A novel suspension multiplex immunoassay for the simultaneous specific detection of lung cancer markers in bronchoalveolar lavage fluid (BALF) clinical samples based on fluorescent microspheres having different size and spectrally encoded with quantum dots (QDEM) was developed. The designed suspension immunoassay was validated for the quantitative detection of three lung cancer markers in BALF samples from 42 lung cancer patients and 10 control subjects. Tumor markers were detected through simultaneous formation of specific immune complexes consisting of a capture molecule, the target antigen, and biotinylated recognition molecule on the surface of the different QDEM in a mixture. The immune complexes were visualized by fluorescently labeled streptavidin and simultaneously analyzed using a flow cytometer. Preclinical validation of the immunoassay was performed and results were compared with those obtained using an alternative 3-plex immunoassay based on Luminex xMAP^®^ technology, developed on classical organic fluorophores. The comparison showed that the QDEM and xMAP^®^ assays yielded almost identical results, with clear discrimination between control and clinical samples. Thus, developed QDEM technology can become a good alternative to xMAP^®^ assays permitting analysis of multiple protein biomarkers using conventional flow cytometers.

Lung cancer is the second most common cancer and the leading cause of all cancer deaths worldwide^1^. Screening and early detection of lung cancer are decisive factors that can considerably reduce the mortality. A number of studies have described panels of specific BALF protein markers for early detection of lung cancer and monitoring of treatment efficiency^2^,^3^. Here, we present a novel suspension microarray for multiplexed analysis of three protein markers associated with lung cancer: α-1-microglobulin/bikunin precursor (AMBP), peroxiredoxin 2 (PRDX2), and Parkinson disease protein 7 (PARK7). AMBP is an inter-alpha-trypsin inhibitor that has been reported to be overexpressed in different cancers, including lung cancer^4^,^5^. PRDX2 is involved in cell redox regulation and plays a protective role as an antioxidant. Its level has been found to be elevated in several human cancer cells and tissues. There is evidence that PRDX2 increases the resistance of cancer cells to antitumor treatment^6^. PARK7, also known as protein DJ-1 (DJ-1), is a positive regulator of androgen receptor-dependent transcription and may function as a redox-sensitive chaperone, protecting cells against oxidative stress and death. Several studies have related PARK7 protein to lung cancer, metastasizing, and treatment resistance^7^,^8^.

Enzyme-linked immunosorbent assay (ELISA) developed in 1971 by Engvall, Perlmann, Schuurs, and Weemen^9^,^10^ has become a widely used routine diagnostic method allowing a wide variety of peptides and proteins, including antibodies and hormones, to be detected and quantified. A serious limitation of this method is that it is a single-analyte technology, providing quantitative detection of only one analyte in a sample. Therefore, multiplexed analyte profiling requires relatively large samples to perform several antigen-specific immunoassays. This analysis is time-consuming and expensive. Moreover, ELISA is designed as a solid-state immunoassay, and the use of a planar matrix can restrict immunoassay capacity, sensitivity, and detection quality^11^.

Suspension (or bead-based) microarrays employing fluorophore-encoded polymeric microspheres are currently of particular interest for multiplexed analysis of biological samples in clinical applications. A wide range of different bead-based microarrays have been developed and successfully applied to detection of specific cancer markers^12^,^13^ and nucleic acids^14^, as well as for multiplexed genotyping^15^,^16^,analysis of single nucleotide polymorphisms (SNPs)^17^, allergy testing^18^, identification of pathogens^19^, and the detection of biological warfare agents^20^.The detection principle is based on optical encoding and quantitative analysis of each analyte with an antigen-specific fluorophore-encoded microbead population carrying its unique optical code. Fluorophore-encoded microspheres can be rapidly analyzed by means of classical flow cytometry^21^,^22^. Therefore, Luminex Corporation has developed a bead-based system consisting of microspheres optically encoded with different amounts of two types of classical organic dyes. However, specific spectral properties of organic dyes often require special equipment for optical code readout that is unavailable in clinical routine. Nevertheless, BD Biosciences company have also developed bead-based system for simultaneous detection up to 30 analytes, such as cytokines, chemokines, growth factors, immunoglobulins, and phosphorylated cell signaling proteins in serum, plasma, or tissue culture supernatant samples. The system is based on microspheres optically encoded with different amounts of a single dye, fluorescing in red spectral region.

Quantum dots (QDs), highly fluorescent nanocrystals, represent a novel class of fluorophores which are a promising alternative to organic fluorescent dyes. QDs are nearly 20 times brighter and thousands of times more stable to photobleaching than conventional organic dyes^23^. Moreover, QDs have broad excitation spectra and narrow emission spectra with Stocks shifts larger than 100 nm^24^. These unique optical properties make QDs ideal probes for multiplexed analysis^25^: a single light source can be used to simultaneously excite QDs of different colors, while their emission spectra are clearly distinguishable. Incorporation of QDs into microspheres allows obtaining a large amount of individual spectral codes providing an attractive alternative to the state-of-the-art suspension microarray diagnostic systems^26^.

Recently, we have developed, characterized, and validated a novel microarray consisting of beads encoded with QDs for the simultaneous detection of free and total prostate-specific antigens, serving as markers of prostate cancer in serum samples^27^. The analytical sensitivity and detection efficiency of the system proved to be equivalent to those of the corresponding single-analyte ELISA-based systems. We concluded that this approach could be easily adapted for simultaneous quantitative profiling of different target analytes (including tumor markers) with high analytical sensitivity, detection quality, and efficiency of analysis.

In this study, we designed and developed a novel multiplexed suspension immunoassay system based on QD-encoded microspheres (QDEM) for simultaneous detection of three lung cancer markers (LCMs) in the human BALF samples.

We focused on the comparison of the results obtained in the preclinical validation of the QDEM-based 3-plex immunoassay for LCMs and the alternative Luminex xMAP^®^ bead-based 3-plex immunoassay. Our data show that quantitative multiplex analysis of specific lung cancer markers in BALF by means of both QDEM and Luminex xMAP^®^ immunoassays yielded highly reproducible and reliable results.

The data show that QDEM technology may be considered an alternative to Luminex xMAP^®^ technology for the diagnosis of complex diseases by analyzing multiple protein biomarkers using conventional flow cytometers.

## Results

### Development and technical validation of the QDEM-based 3-plexsandwich immunoassay

**Figure 1:**
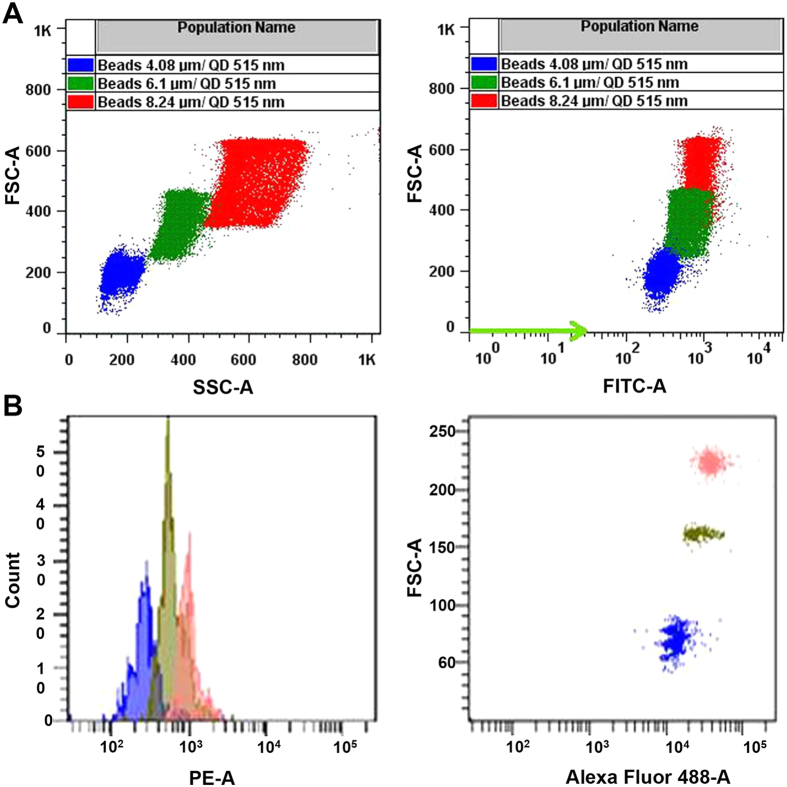
Flow cytometry analysis of quantum dot–encoded microspheres. (**A**) Flow cytometry analysis of three populations of QDEM with sizes of 4.08 μm (blue), 6.1 μm (green), and 8.24 μm (red). (**B**) Representative flow cytometry images obtained by means of the QDEM-based 3-plex immunoassay for lung cancer markers. FSC-A, forward light scattering; SSC-A, side light scattering; PE-A, phycoerythrin channel of flow cytometer.

Melamine formaldehyde (MF) microspheres of different sizes (4.08, 6.1, and 8.24 μm in diameter) were encoded with water-soluble QDs (with an emission maximum at 515 nm) using the layer-by-layer deposition technique to obtain three populations of QDEM, which exhibited intense fluorescence. The populations were easily distinguishable by flow cytometry (Fig. 1).

The Fab fragments of antibodies against three LCMs (AMBP, PRDX2, and PARK7) were covalently coupled to QDEM to obtain three suspension capture antibody systems (QDEM4,08-AMBP, QDEM6.1-PRDX2, and QDEM8.24-PARK7) suitable for 3-plex analysis. In order to avoid nonspecific binding, the capture antibody systems were incubated with 1% BSA. To perform the analysis, the immunodiagnostic complex consisting of a capture antibody, an antigen, and a detection antibody had to be assembled on the QDEM surface. Therefore, QDEM coupled to capture Fab fragments were sequentially incubated with all elements of the immunodiagnostic complex according to the following scheme: (1) the clinical sample (containing the antigen of interest) or the corresponding recombinant protein; (2) biotinylated Fab fragments against each LCM; (3) streptavidin R-Phycoerythrin conjugate (SAPE). Formation of the immunodiagnostic complex was detected in a flow cytometer through colocalization of two fluorescent colors: green (according to fluorescence of QDs with an emission maximum at 515 nm) and orange (according to fluorescence of SAPE with an emission maximum at 578 nm). Hence, the presence and concentration of each LCM in BAL samples were determined by the fluorescent signal of SAPE.

Technical performance of the 3-plex immunoassay was also assessed prior to the preclinical validation with biological samples (Table 1). The intra-assay variation was within the acceptable range (<15%) for the quantification of each marker, the inter-assay variation was <15% for the PARK7 marker, those for AMBP and PRDX2 being slightly beyond the acceptable range (<17%, Table 1). The LoQ was similar for quantification of each marker, ranging between 7 and 10 ng/ml.

### Preclinical validation of the QDEM-based 3-plex immunoassay

Preclinical validation of the QDEM-based multiplexed immunoassay was performed, in which the standard curve with the recombinant protein was included, as well as 10 control and 42 lung cancer BAL samples. The fluorescence intensity data were statistically analyzed.

**Figure 2:**
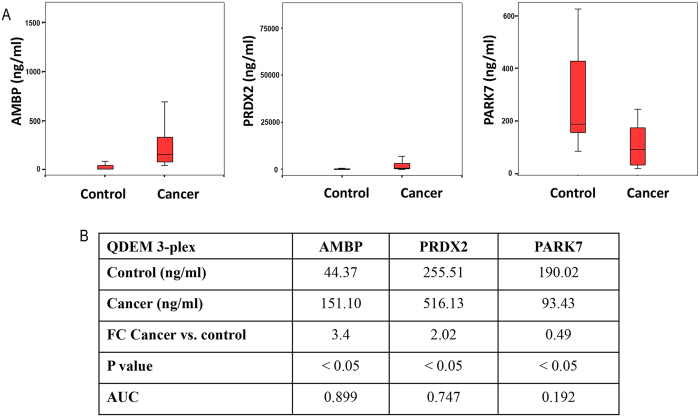
Detection of AMBP, PRDX2, and PARK7 lung cancer markers in bronchoalveolar aspirate lavage samples by means of the QDEM-based 3-plex immunoassay. (**A**) Concentrations of the markers in the control and cancer samples. (**B**) Results of univariate analysis: the marker concentrations interpolated to the standard curve, the FCs for the comparison of cancer and control samples, and the *P* and AUC values. AMBP, α-1-microglobulin/bikunin precursor; AUC, area under the curve; PRDX2, peroxiredoxin 2; PARK7, Parkinson disease protein 7; QDEM, quantum dot–encoded microspheres.

Figure 2 shows the results of AMBP, PRDX2, and PARK7 analyses in the 3-plex immunoassay. Higher concentrations of AMBP and PRDX2 markers were found in the lung cancer group compared to the control group (with a fold change (FC) of 3.4 and 2.0, respectively), whereas PARK7 concentration was lower (FC = 0.49). The differences for the three markers were statistically significant. The AUC values were 0.899, 0.747, and 0.192 for AMBP, PRDX2, and PARK7, respectively, which significantly differed from the threshold value of 0.5.

**Figure 3:**
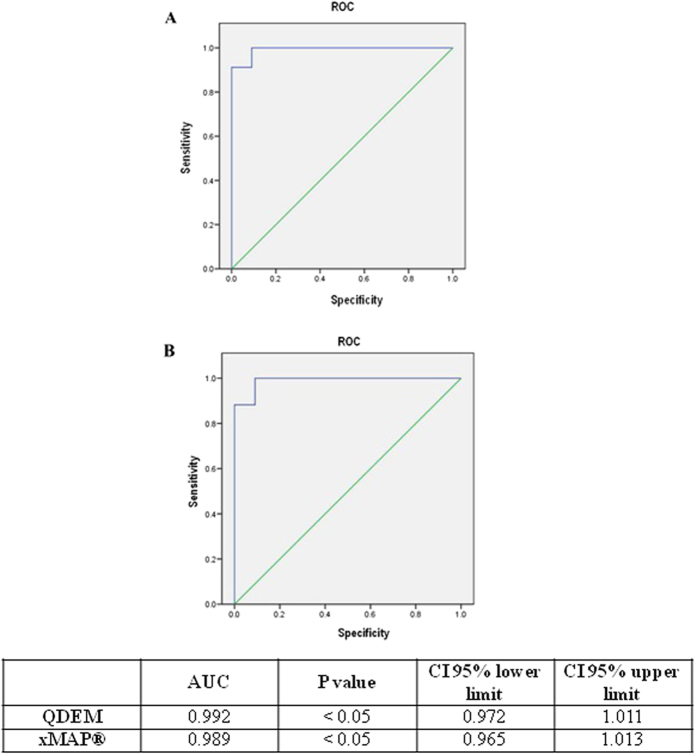
Multivariate analysis of the QDEM-based (**A**) and xMAP^®^-based (**B**) 3-plex immunoassays in bronchoalveolar aspirate lavage samples: receiver operating characteristics of cancer vs. control samples in the model including AMBP, PRDX2, and PARK7 markers. Table shows the results of multivariate comparison of cancer and control samples. AUC, area under the curve; CI, confidence interval; ROC, receiver operating characteristics.

Multivariate analysis yielded a statistically significant model with the AUC value (0.992) exceeding the individual AUC value of each marker analyzed in the 3-plex immunoassay (Fig. 3), thereby improving the discrimination between control and cancer samples.

### Development and technical validation of the xMAP^®^ bead-based 3-plex sandwich immunoassay

We used three populations of fluorescently encoded microspheres: Bead041, Bead016, and Bead048. In order to compare QDEM and xMAP^®^ technologies, the same 3-plex immunoassay for the quantification of AMBP, PRDX2, and PARK7 lung cancer markers was developed for xMAP^®^. For this purpose, the capture Fab fragments against these markers were coupled to fluorescent beads, following the manufacturer’s instructions. The technical performance of this 3-plex immunoassay met the established criteria (Table 1).

### Preclinical validation of the xMAP^®^ bead-based 3-plex immunoassay

A parallel preclinical validation of the 3-plex immunoassay employing the xMAP^®^ technology was performed for the quantification of AMBP, PRDX2, and PARK7 markers in the same 52 BAL samples that were used for the QDEM-based analysis.

**Figure 4:**
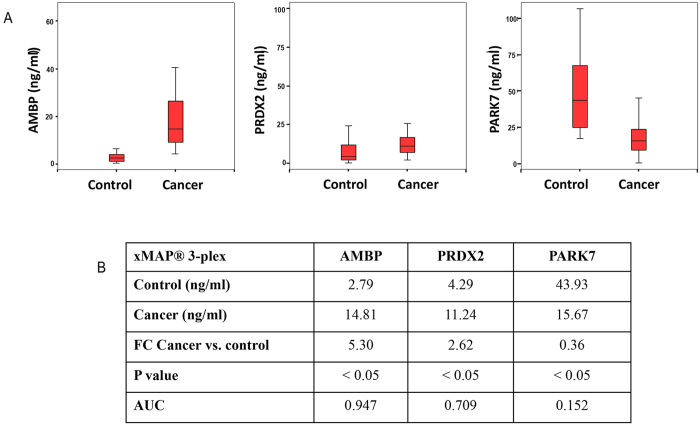
Detection of AMBP, PRDX2, and PARK7 lung cancer markers in bronchoalveolar aspirate lavage samples by means of the xMAP^®^-based 3-plex immunoassay. (**A**) Concentrations of the markers in the control and cancer samples. (**B**) Results of univariate analysis: the marker concentrations interpolated to the standard curve, the FCs for the comparison of cancer and control samples, and the *P* and AUC values. AMBP, α-1-microglobulin/bikunin precursor; AUC, area under the curve; PRDX2, peroxiredoxin 2; PARK7, Parkinson disease protein 7.

As in the QDEM-based analysis, the concentrations of AMBP and PRDX2 markers in the lung cancer group were found to be higher than in the control group (FCs of 5.30 and 2.62, respectively), whereas PARK7 concentration was lower (FC = 0.36). In this xMAP^®^ bead-based analysis, the differences for the three markers were also statistically significant. The AUC values were 0.947, 0.709, and 0.152 for AMBP, PRDX2, and PARK7, respectively, differing significantly from the threshold value of 0.5 (Fig. 4).

Multivariate analysis yielded a statistically significant model with the AUC value (0.989) exceeding the individual AUC value of each marker analyzed in the 3-plex immunoassay (Fig. 3), thereby improving the discrimination between the control and cancer samples.

### Comparison of the results of preclinical validation of the QDEM-based and Luminex xMAP^®^-based immunoassays

As seen from Table 2, the preclinical validation of the QDEM-based and Luminex bead-based 3-plex immunoassays for AMBP, PRDX2, and PARK7 markers as techniques for the diagnosis of lung cancer yielded practically identical results for both of them. Quantification of the three markers analyzed makes it possible to reliably differentiate between the control and lung cancer groups. In addition, the multivariate model, which takes into account the contributions of all the markers, yields AUC values higher than that obtained for each marker alone, thus improving the discrimination.

## Discussion

Recently, we designed and evaluated a novel diagnostic suspension microarray based on QD-encoded microspheres for simultaneous detection of two prostate cancer markers in human clinical serum samples^27^. In addition to the obvious advantage of multiplexing, the designed QD-based suspension system exhibited excellent analytical characteristics and diagnostic efficiency, as well as a high quality of analysis, comparable to those of the conventional “gold-standard” solid-state single-analyte clinical technology.

In comparison with solid-state immunoassays, such as ELISA, suspension microarrays are characterized by a fast binding kinetics due to free motion of microparticles in three dimensions^28^. A set of microparticles with different spectral codes may be used for simultaneous detection of multiple markers in a single biological sample, which can considerably simplify and accelerate the analysis.

The Luminex xMAP^®^ system is based on 5.6-μm polystyrene microspheres (or beads) encoded with two fluorophores (red and infrared) incorporated into them at ten different concentrations, which, in theory, provides ~100 possible combinations. The spectral code of individual populations of microspheres is formed by different amounts of these two dyes. The theoretically possible number of codes (C) is described by the equation C = N^m^ – 1, where N is the number of intensity levels and m is the number of colors^22^. In reality, the number of spectral codes is limited by many experimental factors: reproducibility of the doping process, compatibility of the dyes with the solvent and the polymer material of the beads, the spectral overlap, radiative and/or nonradiative energy transfer between dyes, etc^26^. If the dyes have different excitation wavelengths, then multiple excitation lasers are required, which makes decoding more complicated and expensive. In contrast, due to unique spectral properties of QDs (broad excitation spectra and narrow, symmetric emission spectra), a single light source is enough for excitation of QDs of different colors. In addition, different sizes of beads (microspheres) may be used to increase the number of populations. In this study, we prepared microspheres of three sizes (4.08, 6.1, and 8.24 μm in diameter) encoded with a single type of QDs; the populations were easily distinguishable by flow cytometry analysis (Fig. 1).

The statistical results of the preclinical validation of the QDEM and xMAP^®^ 3-plex immunoassays detecting AMBP, PRDX2, and PARK7 markers as tools for diagnosis of lung cancer were practically identical. Both systems displayed a high quality of discrimination between control and clinical samples, as estimated by multivariate ROC analysis (Fig. 3A).

In these preliminary experiments, we did not aim at achieving a high analytical sensitivity at low concentrations of the markers; our purpose was to assess the diagnostic efficacy and reliability of the designed technology and validation of the array in appropriate concentration ranges for both approaches. Although the estimations of absolute concentrations obtained using the QDEM and xMAP^®^ technologies differed, mainly due to poor fit of the lower zone of the standard curve for QDEM immunoassays, the overall preclinical results of serum sample analysis were highly reproducible and reliable.

Thereby, we adapted the designed QDEM technology for simultaneous detection of three lung cancer markers in human serum and compared it with the alternative Luminex xMAP^®^ suspension immunoassay encoded with classical organic fluorophores. The statistical results of the preclinical validation of the QDEM and xMAP^®^ systems as diagnostic tools for detecting lung cancer markers were practically identical, with clear discrimination between control and clinical samples. The designed detection approach can be easily modified to fit different target analyte profiles, which ensures its versatility. The use of QDs for optical encoding of microspheres have the potential to improve the multiplexing of the assay and can also simplify data processing and the configuration for label excitation and signal decoding. However, high cost of QDs remains a major obstacle to their wide use in clinical analysis for the moment. Actually, high cost of QDs is mainly caused by complicated procedures and specific compounds for their stabilization and solubilization, rather than their own composition and synthesis. He hope that difficulties related to QDs stabilization and solubilization will be solved at the nearest future, that will make QDs more accessible.

Reported results obtained using QDEM-based immunoassays have proved to be very promising and show the potential of this technology as a good alternative tool for the screening and diagnosis of complex diseases by analyzing multiple protein biomarkers using conventional flow cytometers.

## Methods

### Preparation of quantum dot–encoded microspheres (QDEM)

Carboxylated melamine formaldehyde (MF) resin microspheres (Microparticles GmbH, Germany) 4.08, 6.1, and 8.24 μm in size were used as matrix cores for preparation of QDEM. CdSe/ZnS core/shell QDs with an emission wavelength of 515 nm, synthesized by the organometallic colloidal method as described earlier^29^, were kindly provided by Dr. Samokhvalov (Laboratory of Nano-Bioengineering, Moscow Engineering Physics Institute, Moscow, Russia). The QDs were solubilized with carboxyl- and sulfhydryl-terminated derivative of polyethylene glycol carboxy-PEG12-thiol (PEG) (Thermo Fisher Scientific, Villebon-sur-Yvette, France)^30^. Labeling of the microspheres with the QDs was carried out using the layer-by-layer deposition technique^27^. Briefly, the microspheres were alternately incubated in 1 mg/mL aqueous salt solutions of positively and negatively charged polyelectrolytes, poly(allylamine hydrochloride) (PAH) and poly(sodium 4-styrenesulfonate) (PSS), with three washing steps after each incubation. Incubation in aqueous solutions of negatively charged QDs was performed between the depositions of positively charged PAH layers, so that QDs were embedded between PAH layers. Finally, a poly(acrylic acid) (PAA) layer was deposited onto QDEM in order to introduce carboxyl groups required for subsequent coupling of antibodies to QDEM.

The resultant microspheres were transferred to round-bottom test tubes and analyzed with a FACSCantoII flow cytometer (Becton Dickinson, Franklin Lakes, NJ, USA) operating under a 488-nm argon laser excitation to characterize the optical properties and size distribution of the microsphere populations. A minimum of 5,000 events per sample were recorded. The microparticle size distribution was estimated by measuring the side and forward light scatter (SSC-A and FSC-A dot plots). The green (515 nm, optical codes) fluorescence intensities of each microsphere population associated with QD fluorescence were analyzed in the FITC-A channel. The data were processed using the FACSDiva software (Becton Dickinson, Franklin Lakes, NJ, USA).

### Coupling of antibodies to QDEM

QDEM of different size types (4.08, 6.1, and 8.24 μm; 5 . 10^7^ of each) were washed and resuspended in PBS, pH 6.2. Then, the QDEM were activated by adding 10 μL of 50 mg/mL *N*-Hydroxysulfosuccinimide (Sulfo-NHS, Thermo Fisher Scientific, Villebon-sur-Yvette, France) and 10 μL of 50 mg/mL 1-Ethyl-3-(3-dimethylaminopropyl)carbodiimide (EDC, Thermo Fisher Scientific, Villebon-sur-Yvette, France) aqueous solutions, gently mixed in a vortex mixer, and incubated for 20 min at room temperature with gentle mixing. The activated QDEM were washed by microcentrifugation and resuspended in 1 mL of MES, pH 6.0. Then, 500 μg of Fab fragments of antibodies against three LCMs (AMBP, PRDX2, and PARK7) were added to suspensions of 4.08-, 6.1-, and 8.24-μm QDEM, and the suspensions were incubated for 2 h with mixing (by rotation) at room temperature. Coupled microspheres were washed by microcentrifugation three times and finally resuspended in PBS (pH 7.4) with 1% bovine serum albumin (BSA). Coupled QDEM were stored at 2–8 °C in the dark.

### Study samples

For preclinical validation of biological markers previously identified by Progenika Biopharma for lung cancer diagnosis, BALF samples were used. All subjects participating in the study were enrolled by Dr. Rafael Zalacain and Dr. Guillermo López Vivanco at the Hospital Universitario de Cruces (Barakaldo, Spain). The study protocol (code PU-EF) was approved by the ethical committee of Euskadi (CEIC-E, Spain), and all the patients signed an informed consent.

All procedures followed were in accordance with the international ethical standards, in particular the Helsinki declaration of 1975 as revised in 2008, and with the Good clinical practice standards, in particular the 14/2007 law of Biomedical Investigation.

Healthy donors and patients with suspected lung cancer who were finally diagnosed with another respiratory disease (bronchitis or tuberculosis) constituted the control group. Patients with either small cell lung cancer or non-small cell lung cancer at different clinical stages were included. In total, samples from 10 control subjects and 42 lung cancer patients were used in the study (Table 3). Most collected samples belonged to men (88% of male patients vs. 12% of female ones). The age of the enrolled subjects was in the range of 29–82 years, with a mean value of 62 years. In the control group, 80% of the subjects were men, and the mean age was 53 years (ranging between 29 and 69 years), whereas in the lung cancer group, 90% of the subjects were men, and the mean age was 65 years (ranging between 46 and 82 years).

BALF specimens were collected during bronchoscopy for routine diagnostic purposes, by flushing the airways with saline to harvest the surrounding cells. After centrifugation at 3,000 rpm for 15 min at 4 °C, supernatants were frozen and stored at −80 °C until analysis. The diagnosis was confirmed by anatomopathological examination.

### Antibodies and recombinant proteins

In order to obtain sandwich immunoassays, customized antibodies were developed by AbDSerotec (a Bio-Rad company, Puchheim, Germany) using the HuCAL recombinant antibody technology (Human Combinatorial Antibody Library). Pairs of Fab fragments of monoclonal antibodies against AMBP, PRDX2, and PARK7 lung cancer markers, as well as recombinant proteins for plotting standard curves, were obtained.

### Development and analysis of the QDEM-based immunoassay

QDEM-based sandwich immunoassays for the three LCMs were developed to be used in combination with flow cytometry.

For obtaining a single-marker assay, 50 μL of each suspension of the capture Fab fragment coupled to QDEM (QDEM–Fab) diluted in phosphate buffer saline (PBS)–Chemiblocker buffer (Millipore, Billerica, MA, USA), which contained 20,000 QDEM, was mixed with 50 μL of BAL or recombinant protein sample in a well of a 96-well Millipore filter plate (Millipore, Billerica, MA, USA) and incubated for 1 h on a plate shaker at room temperature in the dark. After three washing steps with 200 μL of PBS–Chemiblocker buffer, 50 μL of the detection antibody solution previously biotin-labeled using the EZ-Link™ Sulfo-NHS-Biotin reagent (Thermo Fisher Scientific, Villebon-sur-Yvette, France) following the manufacturer’s instructions were added, and the mixture was incubated under the same conditions. After washing, 50 μL of PhycoLink^®^ streptavidin-R-phycoerythrin (SAPE) (PJRS20, Prozyme, Hayward, CA, USA) was added, and the mixture was incubated for 30 min. Finally, after the washing steps, the mixture in each well was resuspended in PBS and transferred to an Eppendorf test tube to be placed into a FACS system (FACSCanto A, Becton Dickinson, Franklin Lakes, J, USA). Detection was based on measuring fluorescence at the QD and R-phycoerythrin emission wavelengths, as well as the size of the microspheres. The median fluorescence intensity (MFI) values were obtained for each sample quantifying 200 QDEM. The LCM concentration was determined by interpolating the MFI values into the standard curve using the SigmaPlot 11 software (Systat Software Inc, San Jose, CA, USA).

For the development of a 3-plex immunoassay, the same procedure was performed, except that the reagents for the quantification of the three biomarkers were added into a single well. For the quantification of the three proteins identified by Progenika Biopharma as differentially expressed in lung cancer, the BALF dilution, capture antibody, detection antibody, and SAPE concentration were optimized for each candidate biomarker (Table 4).

The immunoassays were also validated. Standard curve fit estimated by backcalculation of standards (the difference between the actual and interpolated concentrations should be <20% for at least six out of seven standards), intra-assay variation (coefficient of variation (CV) < 15%), inter-assay variation (CV < 15%), and the limit of quantification (LoQ) were determined. Technical validation of immunoassays was performed with a 7-point standard curves (Supplementary). For the validation of clinical samples an additional standard curve point was added in the lower zone of the curve, obtaining 8-point standard curves, to improve lower concentration data extrapolation of the biomarkers in the biological samples.

### Development and analysis of the xMAP^®^-based immunoassay

The xMAP^®^ Technology (Luminex Corp., Austin, TX, USA) was used as the “gold-standard” technique for the comparison of LCM validation results obtained with the use of the QDEM technology. Each single and 3-pleximmunoassay was obtained as described above for the QDEM technology (see Table 4 for optimized immunoassay conditions), with the exception that, in the xMAP^®^ technology, capture Fab fragments were coupled to fluorescent beads (Luminex Corp., Austin, TX, USA), following manufacturer’s instructions, and 5,000 beads per well were added.

After the last washing steps, the mixtures were resuspended in Sheath Fluid (Luminex Corp., Austin, TX, USA) and 100 beads per sample read in a Luminex 200 Total System. The median fluorescence intensity (MFI) values were obtained by measuring the fluorescence of each sample.

### Statistical analysis

In order to compare the data on the control and lung cancer groups, two statistical analyses were performed using the SPSS 15 software (SPSS Inc., Chicago, IL, USA).

First, univariate analysis was performed for each assay of the 3-plex immunoassay system. After using the Levene and Kolmogorov–Smirnov tests to assess the suitability of a parametric or a non-parametric analysis, the non-parametric Mann–Whitney U test was used to calculate the P values (the differences between the median values were taken to be significant at P < 0.05).

Second, multivariate logistic regression (the enter method) was used in order to assess how each of the three markers of the multiplexed immunoassay fitted the statistical model. The method included the receiver operating characteristic (ROC) curve and the area under the curve (AUC) values (threshold, 0.5).

## Additional Information

**How to cite this article:** Bilan, R. *et al*. Quantum-dot-based suspension microarray for multiplex detection of lung cancer markers: preclinical validation and comparison with the Luminex xMAP^®^ system. *Sci. Rep.* **7**, 44668; doi: 10.1038/srep44668 (2017).

**Publisher's note:** Springer Nature remains neutral with regard to jurisdictional claims in published maps and institutional affiliations.

## Supplementary Material

Supplementary Information

## Figures and Tables

**Table 1:** Technical performance of the QDEM-based and xMAP^®^-based 3-plex immunoassays for quantification of AMBP, PRDX2, and PARK7 lung cancer markers.

Immunoassay	Standard curve fit	Intra-assay variation	Inter-assay variation	LoQ (ng/ml)
QDEM 4.08-AMBP	5 out of 7	3%	17%	10
QDEM 6.1-PRDX2	5 out of 7	5%	17%	9
QDEM 8.24-PARK7	5 out of 7	5%	10%	7
Bead041-AMBP	6 out of 7	4%	10%	7
Bead016-PRDX2	6 out of 7	10%	13%	3
Bead048-PARK7	6 out of 7	3%	12%	1

AMBP, α-1-microglobulin/bikunin precursor; LoQ, limit of quantification; PARK7, Parkinson disease protein 7; PRDX2, peroxiredoxin 2; QDEM, quantum dot encoded microspheres.

**Table 2:** Comparison of the statistical results of the preclinical validations of the 3-plex immunoassays based on the QDEM and Luminex xMAP^®^ technologies for the diagnosis of lung cancer.

Immunoassay	Marker	Univariate analysis		Multivariate analysis	
		*P* value	AUC	Model AUC	Model *P* value
**QDEM**	AMBP	<0.05	0.899	0.992	<0.05
	PRDX2	<0.05	0.747		
	PARK7	<0.05	0.192		
**xMAP**^®^	AMBP	<0.05	0.947	0.989	<0.05
	PRDX2	<0.05	0.709		
	PARK7	<0.05	0.152		

AMBP, α-1-microglobulin/bikunin precursor; AUC, area under the curve; LoQ, limit of quantification; PRDX2, peroxiredoxin 2; PARK7, Parkinson disease protein 7; QDEM, quantum dot encoded microspheres.

**Table 3:** Classification of bronchoalveolar aspirate lavage samples used for the preclinical validation of biological markers for lung cancer diagnosis.

BALF samples	Control			Lung cancer		
	Healthy	Tuberculosis	Bronchitis	Small cell lung cancer	Non-small cell lung cancer	
					Squamous cell carcinoma	Lung adenocarcinoma
**Number of subjects**	8	1	1	9	21	12

**Table 4:** Experimental conditions for quantification of the three proteins identified by Progenika Biopharma as differentially expressed in lung cancer.

Triple immunoassay		n° QDEM/ beads per well	n° QDEM/ beads per read	Capture antibody	Detection antibody (ug/ml)	SAPE (ug/ml)	BAL dilution
**QDEM**	LCM1	20000	200	10 ug per 1 M microspheres	5	5	1/2
	LCM2	20000	200	10 ug per 1 M microspheres	5		
	LCM3	20000	200	10 ug per 1 M microspheres	2.5		
**xMAP**^®^	LCM1	5000	100	50 ug per 5 M beads	5	5	1/2
	LCM2	5000	100	50 ug per 5 M beads	5		
	LCM3	5000	100	50 ug per 5 M beads	2.5		

QDEM, quantum dot encoded microsheres; LCM, lung cancer marker.
