# Supplementary material for: Quantum-dot-based suspension microarray for multiplex detection of lung cancer markers: preclinical validation and comparison with the Luminex xMAP® system
Source: Sci Rep. 2017 Mar 16;7:44668. doi: 10.1038/srep44668 (PMC5353738; doi:10.1038/srep44668)
Supplement: Supplementary Information [file srep44668-s1.docx]

**Supplementary information**

**Quantum-dot-based suspension microarray for multiplex detection of lung cancer markers: preclinical validation and comparison with the Luminex xMAP® system**

**Regina Bilan^1^, Amagoia Ametzazurra^2^, Kristina Brazhnik^1^, Sergio Escorza^2^, David Fernández^2^, María Uríbarri^2^, Igor Nabiev^1,3^*, Alyona Sukhanova^1,3^***

^1^Laboratory of Nano-Bioengineering, National Research Nuclear University MEPhI (Moscow Engineering Physics Institute), 115409 Moscow, Russian Federation, ^2^Department of Research and Development, Progenika Biopharma S.A., Derio, 48160 Spain, ^3^Laboratoire de Recherche en Nanosciences, LRN - EA4682, Université de Reims Champagne-Ardenne, 51096 Reims, France

*alyona.sukhanova@univ-reims.fr or [igor.nabiev@gmail.com](mailto:igor.nabiev@gmail.com)

**
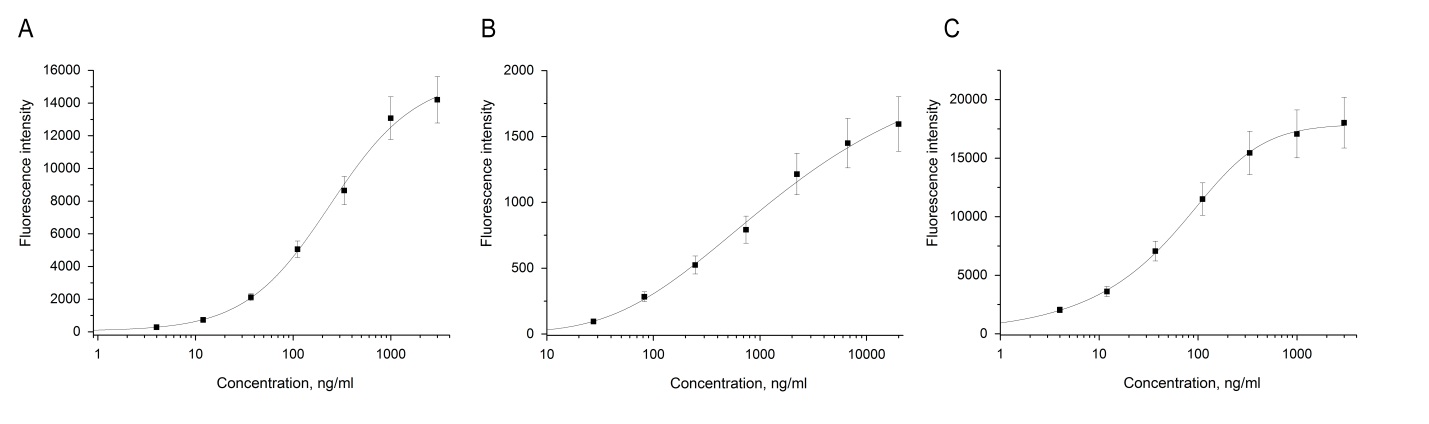
**

Figure S1. Representative images of standard curves of each marker in xMAP®-based 3-plex immunoassay: AMBP standard curve (A), PRDX2 standard curve (B), and PARK7 standard curve (C).


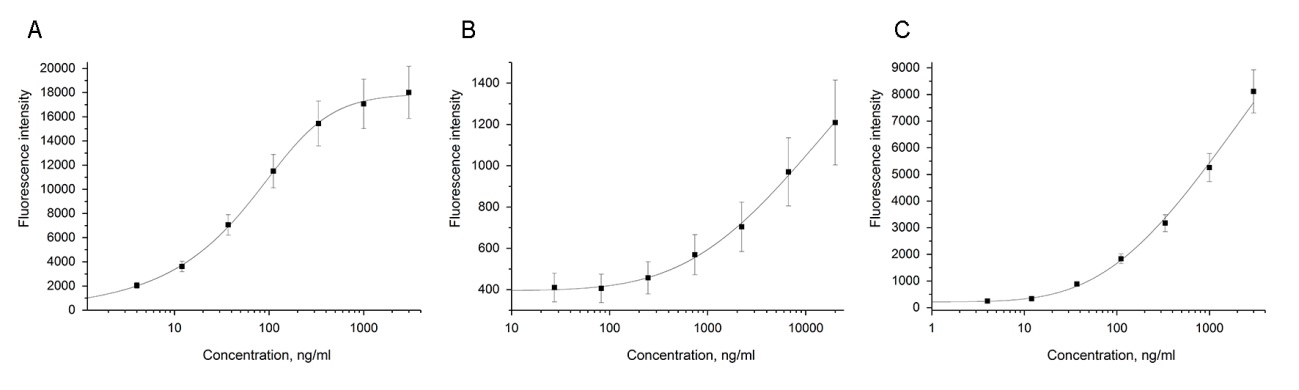


Figure S2. Representative images of standard curves of each marker in QDEM-based 3-plex immunoassay: AMBP standard curve (A), PRDX2 standard curve (B), and PARK7 standard curve (C).
